# Hereditary spastic paraparesis type 18 (SPG18): new *ERLIN2* variants in a series of Italian patients, shedding light upon genetic and phenotypic variability

**DOI:** 10.1007/s10072-024-07423-w

**Published:** 2024-03-01

**Authors:** Ettore Cioffi, Valeria Gioiosa, Alessandra Tessa, Antonio Petrucci, Rosanna Trovato, Filippo Maria Santorelli, Carlo Casali

**Affiliations:** 1 Department of Medico-Surgical Sciences and Biotechnologies, University of Rome Sapienza, Latina, Italy; 2 Department of Neurology and Neurophysiopathology, Azienda Ospedaliera San Camillo Forlanini, Circonvallazione Gianicolense, 87, 00152 Rome, Italy; 3 IRCCS Stella Maris Foundation, Calambrone, Via Dei Giacinti 2, 56128 Pisa, Italy

**Keywords:** Hereditary spastic paraparesis, Amyotrophic lateral sclerosis, Negative dominance, Phenoconversion

## Abstract

**Introduction:**

Hereditary spastic paraparesis (HSP) is a group of central nervous system diseases primarily affecting the spinal upper motor neurons, with different inheritance patterns and phenotypes. SPG18 is a rare, early-onset, complicated HSP, first reported as linked to biallelic ERLIN2 mutations. Recent cases of late-onset, pure HSP with monoallelic ERLIN2 variants prompt inquiries into the zygosity of such genetic conditions. The observed relationship between phenotype and mode of inheritance suggests a potential dominant negative effect of mutated ERLIN2 protein, potentially resulting in a milder phenotype. This speculation suggests that a wider range of HSP genes could be linked to various inheritance patterns.

**Purpose and background:**

With documented cases of HSP loci exhibiting both dominant and recessive patterns, this study emphasizes that the concept of zygosity is no longer a limiting factor in the establishment of molecular diagnoses for HSP. Recent cases have demonstrated phenoconversion in SPG18, from HSP to an amyotrophic lateral sclerosis (ALS)-like syndrome.

**Methods and results:**

This report highlights two cases out of five exhibiting HSP-ALS phenoconversion, discussing an observed prevalence in autosomal dominant SPG18. Additionally, the study emphasizes the relatively high incidence of the c.502G>A variant in monoallelic SPG18 cases. This mutation appears to be particularly common in cases of HSPALS phenoconversion, indicating its potential role as a hotspot for a distinctive SPG18 phenotype with an ALS-like syndrome.

**Conclusions:**

Clinicians need to be aware that patients with HSP may show ALS signs and symptoms. On the other hand, HSP panels must be included in genetic testing methods for instances of familial ALS.

## Introduction

Hereditary spastic paraparesis (HSP) represents a group of genetically heterogeneous diseases that mainly involve the spinal portion of upper motor neurons [1]. HSPs show autosomal dominant, autosomal recessive, X-linked, or mitochondrial inheritance, with over 85 genes loci identified, multiple pathogenic mechanisms [2], and an ample array of neurological and extra neurological accompanying clinical features [3, 4]. From a phenotypical point of view, they are classified into pure or complex [5, 6]. Usually, autosomal dominant HSP (AD-HSP) present with pure phenotypes and are more frequent than autosomal recessive HSP (AR-HSP) [7]. SPG18 stands out as one of the less common forms of HSP documented to date, resulting from either monoallelic or biallelic mutations in the *ERLIN2* gene. ERLIN2 is a lipid-raft-associated protein situated within the endoplasmic reticulum (ER) featuring an SPFH domain [8], and it forms an ERLIN1/2 complex with the closely related ERLIN1 protein; the function of this complex is to bind RNF170, a ubiquitin ligase (E3), which targets activated inositol 1,4,5-trisphosphate receptors (IP3R). These receptors are subsequently ubiquitinated and degraded [8, 9].

SPG18 is in most cases inherited in an autosomal recessive manner (AR-SPG18) [10–15], but few autosomal dominant SPG18 have surfaced (AD-SPG18) [16–19]. The clinical picture of AR-SPG18 usually displays early onset spastic paraparesis, complicated by intellectual disability, motor and speech development delay, multiple joint contractures, seizures [10–12, 14, 17], and in one case progressive juvenile primary lateral sclerosis [13]. AD-SPG18 shows upper motor neuron abnormalities and mild dorsal column abnormalities [16–19]. MRI alterations (i.e., thin corpus callosum—TCC) have been reported in two families [10, 17]. No other laboratory of imaging characteristics has been found. Furthermore, it has been pointed out how some patients with SPG18, both AR and AD, could show phenoconversion to amyotrophic lateral sclerosis (ALS)-like syndromes [20].

Herein, we report two novel *ERLIN2* pathogenic variants found in a series of Italian SPG18 patients. We also review genetic and clinical data from all SPG18 cases described in the existing literature, discussing about genetic variability, phenotypical features, and the importance of zygosity in genetic counselling.

## Materials and methods

### Patients

This multicentric case series study was performed in accordance with the Declaration of Helsinki statements. Written informed consent and ethical approval (CE Lazio) were obtained. In the past 8 years, in a single laboratory, we tested 944 patients with clinical evidence of HSP without a genetic diagnosis, using a multigene targeted resequencing panel (TRP, *n* = 710) or exome sequencing (ES, *n* = 234) or both (*n* = 114) and investigated the coding exons and flanking introns of the genes known to be associated with HSPs [6, 21, 22]. Five patients (4 men; 1 woman) from four families were identified and recruited from three Italian neurology centers (University of Rome Sapienza, Azienda Ospedaliera San Camillo Forlanini, IRCCS Stella Maris Foundation in Pisa). These patients were enrolled in the study and underwent further investigation and analysis. Family and clinical history were collected. All patients underwent neurological examination, clinical cognitive assessment through Montreal Cognitive Assessment (MoCA) [23], and brain MRI (Table 1).

**Table 1:** Clinical, demographic, and radiological details of our patients

Patient/sex	Age at examination	Disease stage at examination	Onset (years)	Duration	Pure HSP	Other features	ID*	ALS phenoconversion	Brain MRI	Mutation
AII.1/M	52	Ambulant-HSP	30	22	Yes	No	No	No	Negative	c.502G > A
AI.2/F	78	Bedridden-ALS	50	28	Yes	No	No	Yes	Negative	c.502G > A
BII.1/M	46	Wheelchair-ALS	25	21	Yes	No	No	Yes	WMA	c.615G > C
CII.2/M	67	Ambulant-HSP	10	50	Yes	Maculopathy	No	No	Negative	c.481C > A + c.866 T > C
DII.3/M	21	Ambulant-HSP	10	11	Yes	Congenital cataract	No	No	Negative	c.374A > G

*HSP* hereditary spastic paraparesis, *ID* intellectual disability, *ALS* amyotrophic lateral sclerosis, *MRI* magnetic resonance imaging

### Molecular and database search

DNA extraction was carried out using peripheral blood lymphocytes obtained from the patients and modalities of next-generation sequencing (NGS) analysis for TRP and ES using methodologies already reported [22, 24]. Search for variants of *ERLIN2* was done using population databases (dbSNP, 1000genome, EVS) and local databases, and their pathogenicity was assessed according to the American College of Medical Genetics and Genomics (ACMG) guidelines [25]. Literature was reviewed using PubMed and Google Scholar, and findings were collected in Table 2.

**Table 2:** Details about clinical, demographical, and radiological findings in all SPG18 cases described in the literature so far

Report	Families	Consanguinity	Cases	Country	Pathogenic variant	Complicated HSP	Pure HSP	ALS phenoconversion	ALS onset	MRI
Al-Yahyaee et al. (2006)	2	Yes	9	Oman	Biallelic locus at 8p12-p11.21 (between markers D8S1820 and D8S532)	Yes: family A (6 casi) with ID and TCC; family B with seizures				Yes: 5 cases in family A with TCC
Yildrim et al. (2011)	1	Yes	13	Turkey	Biallelic c.812_813insAC	yes: EO, progressive, with ID and multiple joint contractures				Neg
Alazami et al. (2011)	1	Yes	5	Saudi Arabia	Biallelic locus at 8p12–8q11.22 (region flanking D8S532 marker)	Yes				Neg
Al-Saif et al. (2012)	1	Yes	4	Saudi Arabia	Biallellic c.499-1G > T	Yes: progressive PLS and ID				Neg
Wakil et al. (2012)	1	Yes	2	Saudi Arabia	Biallelic c.499-1G > T	yes: motor development delay and Speech regression				Neg
Tian et al. (2016)	1	No	1	China	c.538C > T + c.298 + 1G > T		Yes: LO (around 35 y) and long progression			Neg
Morais et al. (2017)	1	No	2	Portugal	Biallelic c.899A > T		yes: EO with long progression			NA
Rydnig et al. (2018)	2	No	17	Norway	Monoallelic c.386G > C		Yes			Neg
Travaglini et al. (2018)	2	No	2	Italy	Biallelic c.866 T > C; c.108-2A > T + c.395C > T		Yes: EO in biallelic, LO in compound heterozygous			Neg
Amador et al. (2019)	4	No	11	France	Monoallelic c.502G > A [p.Val168Met]; biallelic c.899A > T; monoallelic c.374A > G; monoallelic c.926C > T		Yes (2 cases)	Yes (7 cases)	yes (2 cases)	NA
Srivastava et al. (2020)	5	2 yes, 3 no	5	3 Pakistan and Sri Lanka; 1 Italy; 1 Ghana	Biallelic c.861_874dup14; monoallelic c.407 T > G (inherited by maternal isodisomy); monoallelic c.187C > A	Yes: 4 biallelic cases; EO, motor development Delay, speech delay, ID, dystonia, seizures, one with sensorineural hearing loss)	Yes: LO			Yes: 2 biallelic cases with TCC and WMA
Park et al. (2020)	1	No	5	South Korea	Monoallelic c.452C > T		Yes: LO, slow progression			Neg
Chen et al. (2021)	1	No	4	China	Monoallelic c.502G > A [p.Val168Met]		Yes: juvenile-adolescent onset and slow progression			Neg
Present study	4	No	5	Italy	Monoallelic c.502G > A [p.Val168Met]; monoallelic c.615G > C; monoallelic c.374A > G; c.481C > A + c.866 T > C		Yes (3 cases)	Yes (2 cases)		Neg (4 cases); WMA (1 case)

*HSP* hereditary spastic paraparesis, *ALS* amyotrophic lateral sclerosis, *MRI* magnetic resonance imaging, *ID* intellectual disability, *TCC* thin corpus callosum, *EO* early onset, *PLS* progressive lateral sclerosis, *LO* late onset, *WMA* white matter alterations

## Results

### Cases clinical reports

**Fig. 1:**
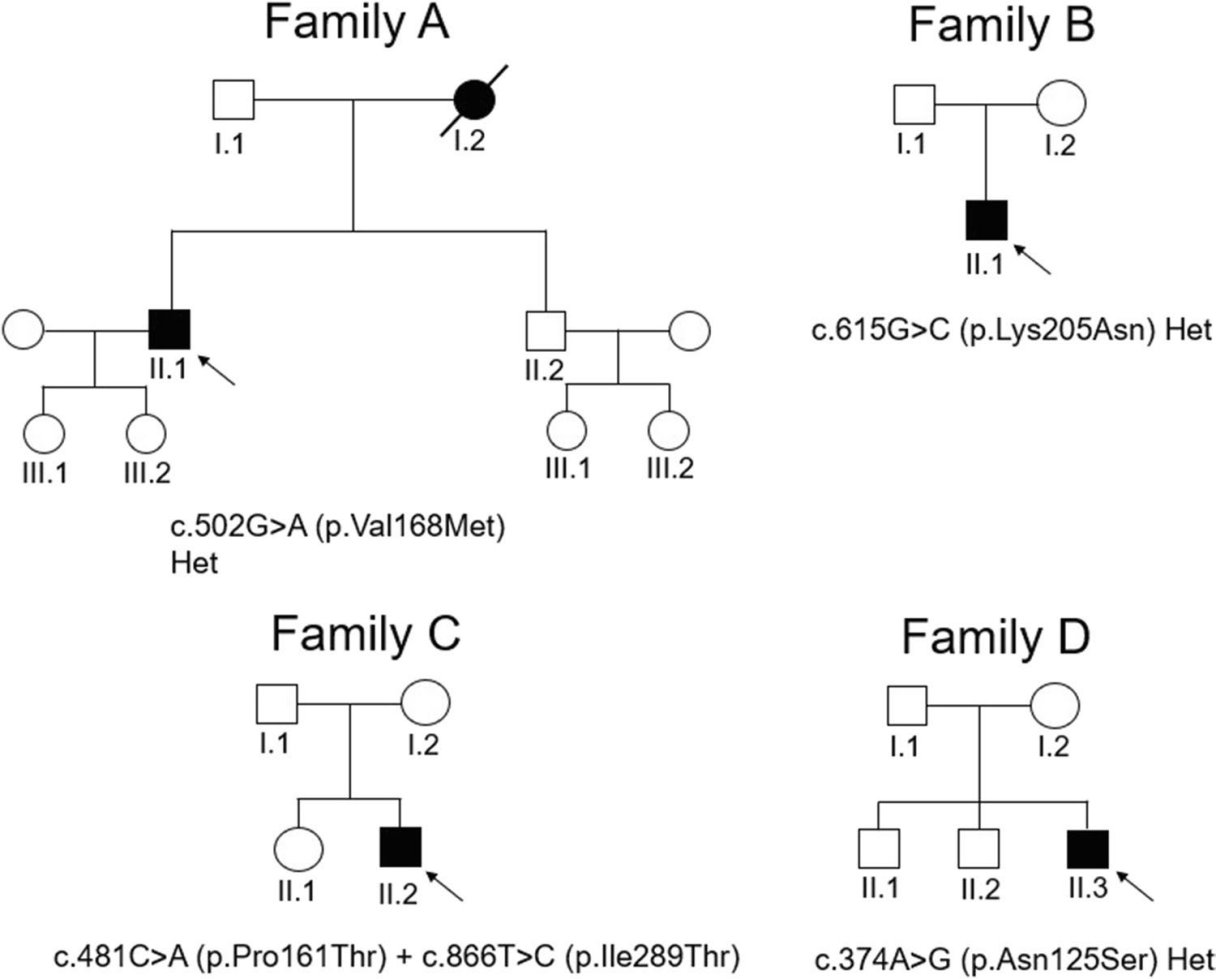
Families’ pedigree. Roman numerals represent the generation. Arabic numerals identify individuals. Arrows indicate the probands. Mutations on the bottom of each pedigree. (Fig. 1 should be placed before the section “Result”)

Clinical, imaging, and laboratory features are summarized in Table 1. Pedigrees are shown in Fig. 1. All families had Italian descent with no reported consanguinity. Three of five individuals had disease onset between 25 and 50 years, while two had juvenile onset (< 10 years). The overall initial manifestation was spastic paraparesis (5/5), with mild dorsal column signs and symptoms (3/5). In one case (BII.1), at the time of the last neurological examination and after 20 years from HSP onset, bulbar and appendicular lower motor neuron signs were identified, suggesting an ALS-like syndrome. Patient AI.2 (mother of AII.1) showed first signs of spastic paraparesis at the age of 50, and her disorder evolved into a rapidly progressive ALS-like syndrome after 27 years (77 years old). She died of ab ingestis pneumonia 12 months later. In one proband, we found maculopathy, while in another one, we observed congenital cataract. No one showed intellectual disability (ID) or extraneurological complications. They all underwent brain MRI: one patient had white matter alterations (WMA). Disease course was slowly progressive (mean 26.4 y at the time of the last examination).

### Molecular findings

Gene testing identified five *ERLIN2* variants, one of which was a compound heterozygous (Fig. 1). The c.502G > A [p.Val168Met], c.866 T > C [p.Ile289Thr], and c.374A > G [p.Asn125Ser] pathogenic variants have already been reported [19, 20, 26]. The other two variants, i.e., the heterozygous c.615G > C [p.Lys205Asn] (CADD score is 24.1) and c.481C > A [p.Pro161Thr] (CADD score is 27.2—found in compound heterozygosis with c.866 T > C [p.Ile289Thr], previously reported [26]) were absent in our in-house databases as well as in population databases (dbSNP, 1000genome, gnomAD) and were classified initially as “hot” VUS and then revised as likely pathogenic according to the ACMG guidelines. Missense variants were indicated as “probably damaging” and “damaging” by multiple in silico predictors (including PolyPhen-2 and SIFT).

## Discussion

We present five previously unreported Italian patients with SPG18. These patients harbored already published disease-associated variants in *ERLIN2*, and in two cases, we detected novel, likely pathogenic variants. This is the 14th report about *ELRIN2*-related motor neuron disease (Table 2). Thus far, a total of 80 cases from 23 SPG18 families (14 biallelic, 9 monoallelic-3 cases presented as sporadic) have been described worldwide [10–19, 26, 27] (Table 2) since the original description by Al-Yahyaee et al. in 2006 [10]. In their work, patients showed complicated AR-HSP (6 cases with intellectual disability and thin corpus callosum, 3 with seizures). *ERLIN2* mutation as causative of SPG18 was first mapped in an AR Turkish family with early onset intellectual disability, motor impairment, and multiple joint contractures in 2011 [11]. Meanwhile, a Saudi family with a mutation in the same locus (flanking the D8S532 marker) received a formal diagnosis. Afterwards, other *ERLIN2* pathogenic variants were identified in additional AR-SPG18 [13–15, 17, 26, 27] families. Data showed a rather uniform phenotype characterized by early onset, complicated and severe spastic paraparesis, usually with a short disease duration. Shortly after, five families with pure HSP harboring monoallelic *ERLIN2* missense mutations had been separately described [16–19]. The clinical manifestation in those families manifests a late onset, progressive and pure spastic paraparesis, with mild dorsal column abnormalities, bearing a strong resemblance to that of other AD-HSP cases. The comparison between AR-SPG18 and AD-SPG18 forms led to the hypothesis that the clinical phenotype of SPG18 may depend on the mode of inheritance, with AD-SPG18 manifesting as a juvenile-adolescent onset pure HSP, whereas AR-SPG18 being a complicated form with earlier onset and more severe course as the likely consequence of a complete loss of function [14]. Presumably, the underlying molecular mechanism in AD-SPG18 is different. Identification of domain-specific mutations in cases from both European and Asian populations makes possible that monoallelic pathogenic variants induce a site-specific dominant negative effect, by disrupting the ERLIN2/ERLIN1 complex, which leads to the more pure and less severe phenotype [28]: functional studies would be required to clarify such issue. To date, only a limited number of HSP loci have been documented to potentially have both AD and AR inheritance patterns, like *REEP2*/SPG72, *ALDH18A1*/SPG9, *KIF1A*/SPG30, and *ATL1*/SPG3A [29–32]. This emphasizes the conundrum in categorizing HSPs solely based on their observable characteristics and that the notion of zygosity no longer serves as a constraining factor when determining a molecular diagnosis in HSP. Indeed, a broader range of HSP genes might be associated with different inheritance patterns, thus having implications for the diagnostic success rate and potentially indicate variations in disease-related characteristics. The existence of different inheritance modes per single disease clearly has implications for genetic counselling, ousting the classic division into AD, AR, X-linked, or mitochondrial patterns. In our series, 4 out of 5 patients have a monoallelic pathogenic variant, and they show a pure HSP. Considering our cases, it appears that AD-SPG18 is as prevalent as AR-SPG18 worldwide. This consideration has implication for counselling and prognosis.

Amador et al. [20] described four pedigrees with an ALS form: two families with monoallelic pathogenic variants (c.502G > A [p.Val168Met] and c.926C > T [p.Ala309Val]), one family with the biallelic pathogenic variant c.899A > T [p.Asp300Val], and a sporadic case with monoallelic pathogenic variant c.374A > G [p.Asn125Ser]. Except for one of the AD families (c.926C > T [p.Ala309Val]), which showed an ALS onset and course without HSP signs, the other cases all exhibited pure HSP-ALS phenoconversion after 20–39 years. After developing ALS-like syndrome, four patients died after a rapidly evolving disease in about 12–18 months. Indeed, from our series, we report two cases of pure HSP-ALS evolution. Patient AI.2, mother of AII.1 with monoallelic pathogenic variant c.502G > A [p.Val168Met], and BII.1 with monoallelic pathogenic variant c.615G > C [p.Lys205Asn], after a long duration of slowly progressive pure HSP (27 years in AI.2, 20 years in BII.1), showed a phenoconversion to an ALS-like syndrome. In one case (AI.2), this condition was rapidly progressive, ultimately leading to death in less than 1 year. To date, nine SPG18 patients with HSP-ALS have been described worldwide. Despite the few cases, we can observe that HSP-ALS conversion is more frequent in AD cases than in AR ones (seven versus two—Tables 1 and 2). Also, phenoconversion seems to occur only in phenotypically pure SPG18 cases (both AD and AR).

A last comment deserves the incidence of the c.502G > A variant, occurring in 11 cases worldwide [19, 20], and only found as a monoallelic gene change. We cannot exclude this variant as a hotspot for a peculiar phenotype of SPG18 with ALS-like syndrome, whereas the multiple genetic background of reported patients makes it unlikely a common ancestor. However, further research is needed to elucidate the disease mechanisms of ERLIN2-related disorders, as well as improved genotype–phenotype correlations.

## Conclusion

We report a large series of Italian SPG18 patients, confirming the prevalence uniformity of both AD and AR forms, as previously described. Phenoconversion of SPG18 into ALS-like syndrome seems to be more frequent in AD-SPG18. We expand the mutational scenario, adding new c.502G > A [p.Val168Met] AD cases with HSP-ALS phenoconversion, thus pointing out the predictive value of this pathogenic variant. The link between HSP and ALS is well known [33–35]. Several HSPs may show ALS-like syndrome during their course, like SPG7 [36], 10 (allelic with Charcot-Marie-Tooth type 2) [37, 38], 11 (allelic with Charcot-Marie-Tooth type 2X) [39, 40], 15 (Kjellin S.) [41], 17 (Silver S.) [42], 20 (Troyer S.) [43]. On the other hand, a few familial ALSs are caused by HSP mutations. The possibility of HSP-ALS phenoconversion has significant clinical implications. Clinicians should be aware of the potential for ALS-like symptoms in individuals with HSP, especially those with mutations in genes that are shared between the two disorders. On the contrary, it is important to bear in mind that, in cases of familial ALS [40], genetic testing should include HSP panels. Recognition of phenoconversion is crucial, as it can inform prognosis, management, counselling, and treatment decisions.

## Data Availability

The authors confirm that the data supporting the findings of this study are available within the article (specifically, Tables 1, 2 and Figure 1). More specific clarifications about data are available, upon reasonable request, from the corresponding author (E.C.).
